# An overview of systematic reviews of complementary and alternative therapies for fibromyalgia using both AMSTAR and ROBIS as quality assessment tools

**DOI:** 10.1186/s13643-017-0487-6

**Published:** 2017-05-15

**Authors:** Rachel Perry, Verity Leach, Philippa Davies, Chris Penfold, Andy Ness, Rachel Churchill

**Affiliations:** 1 University of Bristol, Bristol, England; 2 University of York, York, England

**Keywords:** Fibromyalgia, CAM, Systematic reviews, Overview, ROBIS, AMSTAR

## Abstract

**Background:**

Fibromyalgia (FM) is a chronic, debilitating pain disorder. Dissatisfaction with conventional medicine can lead people with FM to turn to complementary and alternative medicine (CAM). Two previous overviews of systematic reviews of CAM for FM have been published, but they did not assessed for risk of bias in the review process.

**Methods:**

Five databases Medline, Embase, AMED (via OVID), Web of Science and Central were searched from their inception to December 2015. Reference lists were hand-searched. We had two aims: the first was to provide an up-to-date and rigorously conducted synthesis of systematic reviews of CAM literature on FM; the second was to evaluate the quality of the available systematic review evidence using two different tools: AMSTAR (Shea et al. BMC Med Res Methodol 15; 7:10, 2007) and a more recently developed tool ROBIS (Whiting et al. J Clin Epidemiol 69:225-34, 2016) specifically designed to assess risk of bias in systematic reviews. Any review that assessed one of eight CAM therapies for participants diagnosed with FM was considered. The individual studies had to be randomised controlled trials where the intervention was compared to placebo, treatment as usual or waitlist controls to be included. The primary outcome measure was pain, and the secondary outcome measure was adverse events.

**Results:**

We identified 15 reviews that met inclusion criteria. There was low-quality evidence that acupuncture improves pain compared to no treatment or standard treatment, but good evidence that it is no better than sham acupuncture. The evidence for homoeopathy, spinal manipulation and herbal medicine was limited.

**Conclusions:**

Overall, five reviews scored 6 or above using the AMSTAR scale and the inter-rater agreement was good (83.6%), whereas seven reviews achieved a low risk of bias rating using ROBIS and the inter-rater agreement was fair (60.0%). No firm conclusions were drawn for efficacy of either spinal manipulation or homoeopathy for FM. There is limited evidence for topical *Capsicum*, but further research is required. There is some evidence to support the effectiveness of acupuncture for FM, but further high-quality trials are needed to investigate its benefits, harms and mechanisms of action, compared with no or standard treatment.

**Systematic review registration:**

PROSPERO CRD42016035846.

## Background

### Description of the condition

Fibromyalgia (FM) is a chronic pain disorder characterised by widespread pain [1]. It has been described as a ‘central sensitization syndrome’ caused by biological abnormalities in the central nervous system [2] and is often associated with other conditions such as irritable bowel syndrome and depression.

The recently revised FM diagnostic criteria (2011), approved by the American College of Rheumatology (ACR), use a FM Symptom Scale by combining the Widespread Pain Index (WPI) and Symptom Severity Scale (SS) (Wolfe et al. 2011 [3]). The WPI assesses 19 general body areas for pain occurring in the preceding 2 weeks. The severity of the person’s fatigue, unrefreshed waking, cognitive symptoms and general somatic symptoms are rated by the SS for a score ranging from 0 to 12 [3].

FM is reported to affect between 1 and 4% of the population [4]. The use of the new criteria has reduced the gender ratio form 7:1 to 2:1 female to male ratio, which is similar to other chronic pain conditions [5].

FM can develop at any age, including childhood, and there does not appear to be any variation in prevalence with regard to country, culture or ethnic group. Surprisingly, there does not appear to be any variation in industrialised/non-industrialised countries [6].

### Conventional treatments

Medication is currently the main form of treatment; there is strong evidence of an effect for several drugs like antidepressants (e.g. *amitriptyline*) and muscle relaxants (e.g. *cyclobenzaprine)* [7, 8]. However, adverse effects of medication are frequently experienced [9–12]*.* FM is difficult to treat within primary care, and people with FM often turn to complementary and alternative medicine (CAM) therapies; therefore, it is a condition that has received much attention from CAM researchers [13]. Prior research has found that around 90% of people with FM have used at least one form of CAM to manage their symptoms [14–17].

### Description of the interventions

CAM has been defined as ‘…diagnosis, treatment and/or prevention which complements mainstream medicine by contributing to a common whole, by satisfying a demand not met by orthodoxy or by diversifying the conceptual frameworks of medicine’ (Ernst et al.) ([18], p. 506). This review focuses on eight common CAMs which have featured in several CAM surveys [19–21]: acupuncture, hypnotherapy, homoeopathy, osteopathy, chiropractic, herbal medicine, reflexology and aromatherapy (see Appendix 1 for further details on each therapy).

### Why it is important to do this overview

There are two main aims within this overview. The first is to update the synthesis of reviews of CAM literature on FM and establish what evidence is currently available with regard to the efficacy of several CAM practices used in its treatment. As systematic reviews (SR) are often considered the least biased source of evidence to evaluate the efficacy of a particular intervention, this overview will focus on SRs for FM.

The second aim is to provide a robust assessment of the evidence in this area using two complementary quality assessment tools: AMSTAR [22] and ROBIS [23].

### Previous overviews of reviews

Taking a look at previous overviews from the last 5 years, in 2012, Terry et al.’s [1] overview of reviews of CAM for FM identified five systematic reviews. The reviews found some evidence of beneficial effects for acupuncture, homoeopathy, hydrotherapy and massage, whilst no evidence for therapeutic effects for chiropractic treatment of FM symptoms. However, no quality assessment of the individual reviews was performed.

In 2015, Launche et al. [24] also published a synthesis of CAM for FM reviews. The AMSTAR scale [22] was used to assess the quality of the review. In contrast to our overview, Lauche et al. [24] did not restrict the type of CAM, whereas we restricted to the most common CAMs. In addition, we wanted to apply a more rigorous risk of bias assessment to the systematic reviews identified; AMSTAR focuses on the methodological quality of the reviews rather than risk of bias, so we wanted to compensate for that.

In our overview, all eligible systematic reviews of FM were assessed using both the AMSTAR scale [22] and the ROBIS tool [23]. This will provide an up-to-date and rigorous overview of evidence of CAM for FM.

## Methods

This systematic overview was conducted following a predetermined written protocol registered on the PROSPERO database: registration number, CRD42016035846. To be considered eligible for this overview, reviews were required to meet the following criteria:

- *Type of reviews*—all systematic reviews of randomised controlled trials (RCTs) were included. Quasi-experimental studies were included only if they were assessed alongside RCTs and were in the minority. Systematic reviews of quasi-experimental studies are at higher risk of bias due to lack of random assignment, but we did not want to exclude reviews if the majority of included studies were RCTs. All systematic reviews were included with or without a meta-analysis. The reviews must have searched more than one database and reviewed at least one included CAM treatment for FM. However, reviews that assessed several CAM in the same review were considered if they included at least two of the eight relevant CAMS.
- *Type of participant*—reviews that included RCTs using human subjects diagnosed with FM using standard diagnostic criteria (e.g. ACR criteria) were eligible. No restrictions regarding age, gender, condition duration or intensity were applied.
- *Type of intervention*—reviews of effects of any of the following eight CAM therapies were included: acupuncture, hypnotherapy, homoeopathy, osteopathy, chiropractic, herbal medicine, reflexology and aromatherapy. Reviews that included multiple CAM therapies were also included, as long as the CAM therapies were not used in combination. Reviews of complex systems of combinations of a range of therapeutic modalities such as Traditional Chinese medicine (TCM) were excluded as it would be too difficult to establish the separate effects of the individual aspects of this combined approach.
  Reviews that only assessed CAM therapies used as an adjunct therapy to conventional medicine were excluded. CAMs that were used in conjunction with other interventions frequently recommended by mainstream healthcare practitioners to treat FM (exercise, patient education, cognitive/behavioural therapies and hydrotherapy) were also excluded. If reviews had also included some trials using additional medication/exercise, these were included, but those particular trials were excluded from the analysis (both narrative and meta-analysis).
- *Type of comparator* ***—***placebo, no treatment, treatment-as-usual or waitlist control groups were permissible as the comparator.
- *Type of outcome* **—**any review that included studies that reported validated measures of pain (e.g. tender point count on palpation, pain intensity, or assessed using a standardised pain measure such as a visual analogue scale (VAS), McGill Pain Questionnaire (MPQ) [25] and Chronic Pain Grade Scale [26]). Other outcomes extracted were adverse events.

### Excluded reviews:

Any reviews that included participants with co-morbidities (e.g. cancer, drug addiction) were excluded. See Table 4 in Appendix 2 for excluded reviews.

**Fig. 1:**
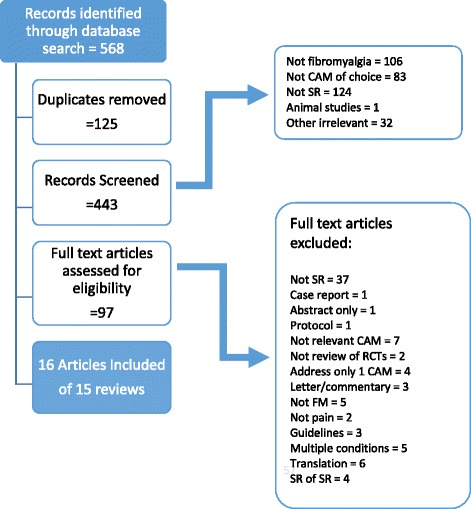
Flow diagram

The following databases were searched from their inception to December 2015: Medline, Embase and AMED (via Ovid), Web of Science and Central via Cochrane library, using a combination of MeSH and key word terms (see Appendix 3 for the search strategy). Conference abstracts/protocols were searched using Web of Science, and authors were contacted to establish progress of their work (see Table 5 in Appendix 2). Reviews had to be published to be included. All titles and abstracts retrieved from the search were assessed for eligibility against the predetermined inclusion criteria by two reviewers (RP, VL). Any review appearing to meet the inclusion criteria based on the abstract was retrieved as a full document. The full-text articles were read in their entirety to assess eligibility by two reviewers (RP, VL) and decisions on inclusion and exclusion recorded (see Fig. 1 for flow diagram). Any disagreements were discussed with a third author (RC). Excluded reviews were recorded alongside reasons (see Table 4 in Appendix 2). Reference lists of all full-text articles were hand-searched for additional studies. We only included English language papers as we did not have access to the translation skills of someone trained in using the ROBIS tool to be able to cross-check the ROBIS tool effectively. Authors of any abstracts/protocols were contacted to establish the status of review.

### Data extracted

Two reviewers (RP, VL) independently extracted data and summarised the review in a characteristic table (see Table 1). Data was extracted from full-text reviews using a standardised data extraction form. The extraction form was piloted prior to starting the overview and refined. Disagreements were resolved through discussion with a third reviewer (RC). Information was extracted from each included review on author, date of review, country, list of studies included in the individual review, intervention and comparator summary, number of participants, diagnosis criteria, meta-analysis results or summary of main between-group results, whether a sensitivity or subgroup analysis was conducted, risk of bias assessment and adverse events.

**Table 1:** Characteristics and results of the included reviews

Author Date Country	Studies included	Intervention group	Comparator group	Type of included study; no. of participants	Length of intervention: no. of sessions: follow up (range)	Diagnosis	Meta-analysis conducted: Y/N main results	Subgroup/sensitivity analysis conducted Y/N	Risk of bias assessment/methodological quality	Safety/ adverse events mentioned
Homoeopathy										
Perry [28] 2010 UK	1. Fisher [35] 2. Fisher [36] 3. Bell [37] 4. Relton [38]	1. *Arnica*, *Bryonia*, *rhus tox* 2. *Rhus tox* 3. Indiv. homoeopathy 4. *Indiv. homoeopathy + TAU* ^a^	1. Placebo pill 2. Placebo pill 3. Placebo pill 4. TAU^a^	RCTs (1 crossover—assessed to first point only) *N* = 163	1. 2× a day for 3 months 2. 3× a day up to crossover at 1 month 3. Daily dose up to crossover at 3 months 4. Daily dose for 22 weeks	No criteria reported	No: 1. Diff. found when remedy is well indicated 2. No diff. found (re-analysis of data) 3. Improvement in TPC and TPP on completers 4. No diff. in FIQ pain scores. In completers, sample greater reduction in MPQ scores (*P* < 0.05)	No	Jadad score plus additional assessment from Cochrane ROB	NR
Boehm [29] 2014 Germany	1. Fisher [35] 2. Fisher [36] 3. Bell [37] 4. Relton [38] 5. Egocheaga [40] CCT	1. *Arnica*, *Bryonia*, *rhus tox* *2. Rhus tox* 3. Indiv. homoeopathy *4. Indiv. homoeopathy + TAU* ^a^ 5. Antihomotoxic injection	1. Placebo pill 2. Placebo pill 3. Placebo pill 4. TAU^a^ 5. Placebo injection	4 RCTs, 1 CCT (plus 13 other types of study NR here) *N* = 183	1. 2× a day for 3 months 2. 3× a day up to crossover at 1 month 3. Daily dose up to crossover at 3 months 4. Daily dose for 22 weeks 5. Injections 2× a week for 8 weeks	ACR criteria	Yes: meta-analysis of 3 RCTs (*n* = 139): effects of homoeopathy on TPC (SMD = −0.42; 95% CI −0.78, −0.05; *P* = 0.03), *I* ^2^ = 0%, compared to placebo Meta-analysis of 2 RCTs and 1 CCT (*n* = 97): effects of homoeopathy on pain intensity (SMD = −0.54; 95% CI −0.97,−0.10; *P* = 0.02 *I* ^2^ = 42%), compared to placebo Homoeopathy had no effect on MPQ scores (2 RCTs)	Yes: (indiv. homoeopathy) no longer an effect on pain intensity *P* = 0.15. Heterogeneity reduced to *I* ^2^ = 13% (*P* = 0.28)	Cochrane ROB	NR
Acupuncture										
Mayhew [43] 2007 UK	1. Martin [52] 2. Assefi [54] 3. Guo [53]^c^ 4. Sprott [50] 5. Deluze [51]	1. EA 2. TCA 3. (i) EA; (ii) DE 4. TCA 5. EA	1. Sham TCA 2. (i) Unrelated TCA for FM; (ii) not acupuncture points; (iii) Sham needling 3. AD, vit. B, oryzanol 4. Sham needling 5. Sham EA	4 RCTs, 1 quasi-RCT *N* = 316	1. 6 sess. over 3 weeks, FU 1, 7 months 2. 24 sess., FU 3, 6 months 3. 28 sess. over 30 days, FU 6 months 4. 6 sess. over 3 weeks, FU 2 months 5. 6 sess. over 3 weeks	ACR criteria	No: 1. FIQ score improved more in TCA gp during study period (*P* = 0.01), at 1 month (*P* = 0.007) but not after 7 months (*P* = 0.24) 2. No diff. between TCA and pooled sham gp 3. Diff. between acupuncture gps and control 4. Number of TP decreased in TCA gp. This was not maintained at 2 months 5. Pain threshold improved by 70% in EA gp v 4%. Pain on VAS also improved more in EA gp	No	Jadad score	Yes
Daya [49] 2007 UK	1. Martin [52] 2. Assefi [54] 3. Singh [96]^c^ 4. Sandberg [97]	1. EA 2. TCA 3. TCA 4. TCA	1. Sham TCA 2. (i) Unrelated TCA for FM; (ii) not acupuncture points; (iii) Sham needling 3.NR—no control arm 4. Crossover	3 RCTs (1 crossover), 1 quasi-RCT *N* = 58 completed	1. 6 sess. over 3 weeks, FU 1, 7 months 2. 24 sess., FU 3, 6 months 3. NR 4. 10–14 sess. over 2–3 months	ACR criteria	No: 1. FIQ *P* = 0.007, 7 months, FU NS (*P* = 0.24) 2. No dif. between TCA and pooled sham gp for pain (*P* > 0.2) or number of pain meds used during active treatment^b^ 3. Pre-post data only 4. TPC = *P* = 0.03; medication intake *P* = 0.03; pain intensity *P* = 0.01	No	van Tulder	Yes
Langhorst [44] 2009 Germany	1. Assefi [54] 2. Deluze [51] 3. Harris [55] 4. Harris [56] 5. Lautensclauger [57] 6. Martin [52] 7. Sprott [50]	1. TCA 2. EA 3. TCA 4. TCA 5. TCA 6. EA 7. TCA	1. (i) Unrelated TCA for FM; (ii) not acupuncture points; (iii) sham needling 2. Sham EA 3. Sham needling 4. Not acupuncture points 5. Sham needling 6. Sham EA 7. Sham needling	7 RCTs *N* = 385	1. 24 sess., FU 3, 6 months 2. 6 sess. over 3 weeks 3. 18 sess. over 13 weeks 4. 9 sess. over 4 weeks 5. 6 sess. over 2 weeks 6. 6 sess. over 3 weeks, FU 1, 7 months 7. 6 sess. over 3 weeks, FU 2 months	6 used ACR 1 used criteria of generalised tendo-myapthia	Yes: pooled analysis of 7 studies (*n* = 242) indicate strong evidence for the reduction of pain (SMD −0.25; 95% CI −0.49 to −0.02; *P* = 0.04, *I* ^2^ = 1%) at post-treatment compared to sham/simulated acupuncture	Yes	Cochrane ROB and van Tulden score	Yes
Martin-Sanchez [45] 2009 Spain	1. Lautenschlauger [57] 2. Deluze [51] 3. Sprott [50] 4. Assefi [54] 5. Harris [55] 6. Martin [52]	1. EA 2. EA 3. TCA 4. TCA 5. TCA 6. EA	1. Sham needling 2. Sham EA 3. Sham needling 4. (i) Unrelated TCA for FM; (ii) not acupuncture points; (iii) sham needling 5. Not acupuncture points 6. Sham EA	6 RCTs *N* = 323	1. 6 sess. over 2 weeks 2. 6 sess. over 3 weeks 3. 6 sess. over 3 weeks, FU 2 months 4. 24 sess., FU 3, 6 months 5. 18 sess. over 13 weeks 6. 6 sess. over 3 weeks, FU 1, 7 months	ACR criteria	Yes: Pain intensity—pooled analysis of 4 studies (*n* = 257) indicated no diff. between gps from baseline: SMD 0.02 (95% CI −0.24 to 0.28). Considerable intra-study homogeneity was in evidence *P* = 0.41, *I* ^2^ = 0%	No	NR	NR
Cao [47] 2013 China	1. Assefi [54] 2. Cao [98] 3. Deluze [51] 4. Gong [61] 5. Hadianfard [62] 6. Harris [55] 7. Harris [59] 8. Jiang [64] 9. Lautensclager [57] 10. Liu [99] 11. Liu [60] 12. Martin [52] 13. Ruan [61] 14. Sprott [50] 15. Targino [65] 16. Yao [63]	1. TCA *2. TA + cupping + AD* 3. EA 4. TA 5. TA 6. TA 7. TA *8. (i) EA + cupping; (ii) EA + cupping + AD* 9. TA 10. TA *11. (i) TA; (ii) TA + Vit B12* 12. EA 13. Moxibustion *14. EA + basic therapy* *15. TA + usual care* 16. TA	1. (i) Unrelated TCA for FM; (ii) not acupuncture points; (iii) sham needling 2. Seroxat (AD) 3. Sham needling 4. Amitriptyline (AD) 5. Fluoxetine (AD) 6. (i) Sham needling; (ii) unrelated TCA; (iii) sham needling in unrelated sites 7. Sham needling 8. Amitriptyline (AD) 9. Sham needling 10. Painkiller (ibuprofen) 11. Amitriptyline (AD) 12. Sham EA 13. Amitriptyline (AD) 14. Sham EA 15. AD + exercise (=usual care) 16. Amitriptyline (AD)	16 RCTs (12 in meta-analysis) *N* = 1081	1. 24 sess., FU 3, 6 months 2. 9 sess. over 4 weeks 3. 6 sess. over 3 weeks 4. Once daily to 2× wkly for 12 weeks 5. 32 sess. over 8 weeks 6. 18 sess. over 13 weeks 7. 9 sess. over 4 weeks 8 (i) 12 sess. over 4 weeks; (ii) every day for 4 weeks 9. 6 sess. over 2 weeks 10. Every day for 2 weeks 11. Every day for 4 weeks 12. 6 sess. over 3 weeks, FU 1, 7 months 13. Every day for 4 weeks 14.4–8 sess. over 2–4 weeks, FU 2 months 15. 20 sess., FU 3, 6, 12, and 24 months 16. Every day for 4 weeks	15 used ACR 1 used IASR criterion	Yes: Change in VAS pain score: no diff. between acupuncture and sham on reducing pain shown in pooled analysis of 7 arms: SMD −0.09 (95% CI −0.32, 0.14) *P* = 0.44 *I* ^2^ = 2% or at post-treatment SMD −0.22, (95% CI −0.51 to 0.07) *P* = 0.13, *I* ^2^ = 26% Pooled analysis of 4 trials showed acupuncture was better than ADs in VAS pain scores: SMD −0.60 (95% CI −0.93 to −0.27, *P* = 0.0004, *I* ^2^ = 22%	Yes	Cochrane ROB	Yes
Deare [48] (Cochrane review) 2013 Australia	1. Assefi [54] 2. Deluze [51] 3. Guo [66]^c^ 4. Harris [55] 5. Harris [59] 6. Harris [59] 7. Itoh [67] 8. Martin [52] 9. Targino [65]	Restricted to acupuncture that penetrated the skin: 1. TCA 2. EA 3. TCA 4. TCA 5. TA 6. TA 7. EA or TPA 8. EA 9. TA + usual care	1. (i) Unrelated TCA for FM; (ii) not acupuncture points; (iii) sham needling 2. Sham EA 3. Amitriptyline 4. (i) Sham needling; (ii) unrelated TCA; (iii) sham needling in unrelated sites 5. Sham needling 6. Sham needling 7. Less acupuncture 8. Sham EA 9. AD + exercise (usual care)	8 RCTs 1 quasi-RCT *N* = 395	1. 24 sess., FU 3, 6 months 2. 6 sess. over 3 weeks 3. 28 sess. over 30 days, FU 6 months 4. 18 sess. over 13 weeks 5. 9 sess. over 4 weeks 6. 9 sess. over 4 weeks 7. 10 sess. over 5 weeks (after 5 weeks) 8. 6 sess. over 3 weeks, FU 1, 7 months 9. 20 sess., FU 3, 6, 12, and 24 months	ACR criteria	Yes: Pain severity using VAS (100-mm NRS, MPI, and MPQ. 6 studies: no diff. between MA/EA and sham in reducing pain: SMD −0.14; 95% CI −0.53 to 0.24, *P* = 0.48. *I* ^2^ = 54%. Reduction in pain (VAS) for those treated with acupuncture compared with no acupuncture at the end of treatment. 1 study: mean diff. (MD) −22.40 points on a 100-point scale; 95% CI −40.98 to −3.82, *P* = 0.02) Short-term benefit of acupuncture over ADs 1 study VAS = −17.3 on a 100-point scale; 95% CI −24.1 to −10.5	Yes	Cochrane ROB	Yes
Yang [46] 2013 China	1. Deluze [51] 2. Martin [52] 3. Harris [55] 4. Wang [100] 5. Guo [66] CCT 6. Guo [101] CCT 7. Wang [102] 8. Guo [53] CCT 9. Targino [65]	1. EA 2. EA 3. TCA 4*. TCA + ALI* 5. TCA 6. *EA with TDP* 7. TCA 8. (i) DE; (ii) EA 9. *TCA + usual care*	1. Sham EA 2. Sham EA 3. (i) Unrelated TCA; (ii) sham needling in unrelated sites 4. Amitriptyline 5. Amitriptyline 6. Fluoxetine 7. Amitryptaline + oryzanol + vit B1 8. (i) Amitriptyline; (ii) amitriptyline *9. AD + exercise (usual care)*	6 RCTs + 3 CCTs *N* = 592	1. 6 sess. over 3 weeks 2. 6 sess. over 3 weeks, FU 1, 7 months 3. 18 sess. over 13 weeks 4.20 days 5. 28 sess. over 30 days, FU 6 months 6. 4 weeks 7. 4 weeks 8. 45 days 9. 20 sess, FU 3, 6, 12, and 24 months	ACR criteria	Acupuncture V sham acupuncture: inaccurate meta-analyses—used control group from Harris (2005) twice Acupuncture V AD at 45 days: inaccurate meta-analyses—used control group from Guo (2010) twice Single studies used for the remaining meta-analyses	Yes: sub group analyses were completed but the meta-analyses were not conducted appropriately	Cochrane ROB	Yes
Chiropractic										
Ernst [73] 2009 UK	1. Blunt [69] 2. Tyers [72]^c^ 3. Wise [70] 4. Panton [71]	1. Chiropractic care 2. *Chiropractic treatment + CES + rugs* 3. *Chiropractic adjustments + soft tissue therapy* *4. Chiropractic + RT*	1. WL 2. *CES + drugs* 3. Ultrasound or no treatment 4. RT only	3 RCTs + 1 quasi-RCT *N* = unclear due to missing information	1. 4 weeks 2. 3× wk for 3 weeks 3. NR 4. 2× a week for 16 weeks	No criteria reported	No: 1. No diffs. on any outcomes 2. 34% pain reduction on VAS v 26% reduction in control, no statistical analysis provided 3. NR 4. No between group diffs. found but no analysis presented	No	Jadad score	No
Herbal medicine										
de Souza Nascimento [75] 2013 Brazil	1. Casanueva [76] 2. McCarty [77] 3. Ware [78] 4. Skrabek [79] 5. Rutledge [80] 6. Ko [81] 7. Lister [82]^c^ 8. Lukaczer [83]^c^	1. Capsaicin (T) 2. Capsaicin (T) 3. Nabilone (O) 4. Nabilone (O) *5. Oil24 (T) + exercise* 6. Oil24 (T) *7. Coenzyme Q10 and ginko (O)* 8. Meta050 (O)	1. TAU 2. TAU 3. Amitriptyline (AD) 4. Placebo *5. Peppermint oil + exercise* 6. Peppermint oil 7. No control gp 8. No control gp	6 RCTs (1 crossover) + 2 observational studies *N* = 475	1. 0.075% 3× a day for 6 weeks, FU at 6 weeks 2. 0.0025% 4× a day for 4 weeks 3. 0.5 to 1 mg for 2 weeks 4. 0.5 to 1 mg over 4 weeks, FU at 8 weeks 5. 3× a week for 12 weeks 6. 1 month 7. 12 weeks 8. 440 mg 3× day for 4 weeks then 880 mg 2× a day for 4 weeks	ACR criteria	No: Capsicum: 1. Improvement in myalgic score, PPT, FSS, FIQ 2. Improvement in sensitivity and pain Nabilone: 3. Similar to amitriptyline on pain rating 4. Decrease in pain in nabilone group 024 oil : 5. Pain score NR 6. Improvements noted on VAS for night pain rating Meta 050: 7. No control gp so no relevant analysis Coe10 and ginko: 8. No control gp so no relevant analysis	No	Jadad and Cochrane ROB	Yes
Multiple cam										
Holdcroft [30] 2003 USA	1. Deluze [51] 2. Feldman [103] 3. Fisher [36] 4. Blunt [69]	Multiple CAM (4 relevant): 1. EA *2. EA + amitryptaline (AD)* 3. *Rhus tox* 4. Chiropractic	1. Sham needling *2. Sham needling and amitriptyline (AD)* 3. Placebo pill 4. TAU (WL control)	4 relevant RCTs *N* = 179	1. 6 sess. over 3 weeks 2. 16 weeks 3. 3× a day up to crossover at 1 month 4. 4 weeks	No formal diagnosis of FMS reported	No: 1. Pain threshold improved by 70 V 4% in the sham acupuncture group. 2. Pain differed between acupuncture and sham group 3 mean number of TP reduced by 25% and pain on VAS improved compared to placebo 4. *P* > 0.05 for chiropractic	No	Consort 22	Yes
Baronowsky [31] 2009 Germany	1. Assefi [54] 2. Deluze [51] 3. Martin [52] 4. Sprott [50] 5. Bell [37] 6. Blunt [69] 7. Gamber [74]	Multiple CAM (7 relevant): 1. TCA 2. EA 3. EA *4. EA + basic therapy* 5. Indiv. homoeopathy 6. Chiropractic 7. Osteopathy	1. (i) Unrelated TCA for FM; (ii) not acupuncture points; (iii) sham needling 2. Sham EA 3. Sham EA 4. Sham needling 5. Placebo pill 6. TAU (WL control) 7. (i) TAU; (ii) moist heat treatment	7 relevant RCTs *N* = 357	1. 24 sess., FU 3, 6 months 2. 6 sess. over 3 weeks 3. 6 sess. over 3 weeks, FU 1, 7 months 4. 6 sess. over 3 weeks, FU 2 months 5. Daily dose up to crossover at 3 months 6. 4 weeks 7. Every week for 6 months		1. No diff. between gps 2. Improvement in treatment group in pain threshold 3. Improvement in FIQ (*P* = 0.01) and MPI (*P* = 0.03) up to 1 month 4. Decrease in TPC compared to usual care but not sham 5. Improvement in TPC and TP pain on palpation compared to placebo 6. No diffs. were found 7. Osteopathy gp better than control in pain threshold in 3 TP (plus some subcategories of various pain scales)	No	Yes: non-standardised quality scale (16 formal criteria)	No
De Silva [32] 2010 UK	1. Fisher [35] 2. Fisher [36] 3. Bell [37] 4. McCarty [77]	Multiple CAM (4 relevant): 1. *Arnica, Bryonia, rhus tox* *2. Rhus tox* 3. Indiv. homoeopathy 4. Capsicum (T)	1. Placebo pill 2. Placebo pill 3. Placebo pill 4. TAU	4 relevant RCTs *N* = 161	1. 2× a day for 3 months 2. 3× a day up to crossover at 1 month 3. Daily dose up to crossover at 3 months 4. 0.0025% 4× a day for 4 weeks	‘Recognised criteria for FM’	No: Homoeopathy: 1. *Rhus tox*—improvement in TPC (*P* < 0.005) 2. Improved pain VAS *P* < 0.05 3. Improvement in TP pain, TPC compared with placebo Capsicum: 4. Improvement in tenderness	No	Jadad score	Yes
Terhorst [33, 34] 2011, 2012 USA	1. Bell [37] 2. Fisher [36] 3. Relton [38] 4. Blunt [69] 5. Gamber [74] 6. Panton [71] 7. Assefi [54] 8. Deluze [51] 9. Harris [55] 10. Itoh [67] 11. Martin [52] 12. Jiang [64] 13. Targino [65]	Multiple CAM (13 relevant): 1. Indiv. homoeopathy 2. *Rhus tox* *3. Indiv. homoeopathy + usual care* ^a^ 4. Chiropractic 5. Osteopathy *6. Chiropractic + RT* 7 TCA 8. EA 9. TCA 10. EA or TPA 11. EA *12. (i) EA + cupping*; *(ii) EA + cupping + AD* *13. TCA + usual care*	1. Placebo pill 2. Placebo pill 3. TAU 4. TAU (WL control) 5. (i) TAU; (ii) moist heat treatment 6. RT only 7. (i) Unrelated TCA for FM; (ii) not acupuncture points; (iii) sham needling 8. Sham EA 9. Sham TCA 10. Less acupuncture 11. Sham EA 12. Amitriptyline (AD) *13. AD + exercise (usual care)*	13 relevant RCTs Acupuncture = 329 Manipulation = 52 Homoeopathy = 131	1. daily dose up to crossover at 3 months 2. 3× a day up to crossover at 1 month 3. Daily dose for 22 weeks 4. 4 weeks 5. Every week for 6 months 6. 4 weeks 7. 24 sess., FU 3, 6 months 8. 6 sess. over 3 weeks 9. 18 sess. over 13 weeks 10. 10 sess. over 5 weeks (after 5 weeks) 11. 6 sess. over 3 weeks, FU 1, 7 months 12. (i) 12 sess. over 4 weeks; (ii) every day for 4 weeks 13. 20 sess., FU 3, 6, 12, 24 months	ACR, Yunus or Smythe criteria	Acupuncture (6/7 studies) A modest treatment effect in favour of acupuncture Spinal manipulation (2/3 studies) Both studies had effect sizes that were in the direction of the treatment group. No overall effect size was given because of the limited number of studies with very small sample sizes. Homoeopathy (2/3 studies) One homoeopathic study favoured the treatment group	No	GRADE	No

Italics = CAM plus another intervention

^a^Usual care—one or more of the following physiotherapy, aerobic exercise, anti-inflammatory drugs, antidepressants

^b^Three sham acupuncture groups combined

^c^Quasi-experimental

*EA* electro-acupuncture, *TCA* Traditional Chinese acupuncture, *MA* manual acupuncture, *TPA* trigger point acupuncture, *ALI* acupoint laser irradiation, *AD* antidepressants, *AI* anti-inflammatory, *TAU* treatment as usual, *FU* follow up, *ACR* American College of Rheumatology, *IASR* International Academy of Soreness Research, *Nabilone* cannabinoid extract, *AEs* adverse events, *TPC* tender point count, *WL* waitlist, *TPP* tender point pain, *TPS* trigger point stimulation, *RCT* randomised controlled trial, *CCT* controlled clinical trial, *ROB* risk of bias, *FU* follow-up, *gp* group, *diffs* differences, *sess.* sessions, *VAS* visual analogue scale, *FIQ* Fibromyalgia Impact Questionnaire, *PPT* pain pressure threshold, *NR* not reported, *SMD* standard mean difference, *MD* mean difference, *MPQ* McGill Pain Questionnaire, *MPI* multi-dimensional pain inventory, *TDP* specific electromagnetic spectrum treatment, *indiv.* individualised, *RT* resistance training

We extracted the mean and standard deviation (SD) of continuous variables and any between-group statistical analyses. We reported the standard mean difference (SMD) and 95% confidence intervals (CI) and results of any tests of heterogeneity reported in the relevant meta-analyses. If ‘pain’ was measured alongside another outcome (e.g. discomfort) and recorded as a single variable, we would extract the data and highlight this in the table and text.

### Data synthesis

Due to the expected overlap of studies and heterogeneity between reviews (particularly with regard to interventions and comparator arms), we conducted a narrative synthesis of the findings rather than pooling of meta-analyses from the included reviews.

#### Assessment of methodological quality/bias of the included reviews

The quality of each systematic review was assessed using both the frequently used and validated AMSTAR tool [22, 27] alongside the newly developed ROBIS tool [23]. AMSTAR is an 11-item tool that has been used frequently to check the quality of a systematic review and determine whether the most important elements are reported (http://www.robis-tool.info). It consists of a series of questions with four possible answers. Each question is not evenly weighted so and although an overall score is sometimes reported, this is not what the tool is intended for. It is frequently used in Cochrane overviews and by the Scottish Intercollegiate Guidelines Network (SIGN). It is intended for reviews that address questions of effectiveness that include just randomised controlled trials (RCTs). However, AMSTAR does not cover some quality items, and each item is not weighted the same; thus, we felt it important to also use the newly developed ROBIS tool.

The aim of the ROBIS tool is to evaluate the level of bias present within a systematic review (http://amstar.ca/About_Amstar.php). This tool assesses the level of bias across four domains: *study eligibility criteria*, *identification and selection of studies*, *data collection and study appraisal* and *synthesis and findings.* Each domain has signalling questions and a judgment of concerns about risk of bias of the domain (low, high or unclear—see Table 6 in Appendix 4). In the final phase, the reviewer makes a judgment about the overall risk of bias. In contrast to AMSTAR, ROBIS has a wider application and is intended for assessing effectiveness, diagnostic test accuracy, prognosis and aetiology. It has an optional phase to assess the applicability of the review to the research question of interest.

Two reviewers (RP, VL) independently assessed each review using both tools. Both reviewers had limited experience of using the ROBIS tool, so a third reviewer who helped develop the tool (PD) was asked to also complete the ratings. Meta-analyses were checked by a statistician experienced in meta-analyses (CP). The inter-rater reliability of overall ratings using each instrument (AMSTAR and ROBIS) was calculated also using the unweighted kappa statistic and percentage agreement. We interpreted cut-offs for Kappa values as <0.20 = poor agreement, 0.21 to 0.40 = fair, 0.41 to 0.60 = moderate, 0.61 to 0.80 = good and 0.81 to 1.00 = very good agreement.

### Deviation from the protocol

In our protocol [PROSPERO CRD42016035846], we said we would not apply any language restrictions; however, it was decided that we would only include English language papers as the ROBIS tool would be a complex tool to ask someone to extract data with.

## Results

### Results of the literature search

The search strategy yielded 568 potentially relevant papers for inclusion. After 125 duplicate titles were removed, 443 remained. Once screened, 98 papers were identified as potentially eligible and full-text copies were retrieved and reviewed by the two reviewers (RP, VL) (see Fig. 1 for flow diagram). From these papers, 15 were included in this overview, and the reasons for excluding articles are presented in Table 4 in Appendix 2. Results of the included studies are presented in Table 1. The summarised AMSTAR scores are presented in Table 2, and the summarised ROBIS scores are presented in Table 3. The justification statements for ROBIS are presented in Table 6 in Appendix 4.

**Table 2:** AMSTAR

Author (date) CAM	A priori design	Two data extractor and consensus?	Comprehensive literature search?	Statement on inclusion of grey literature? Language?	List of included and excluded studies?	Characteristics of studies (tables)	Quality of risk of bias	Scientific quality of the included studies used appropriately in formulating conclusions?	Methods used to combine the findings of studies appropriate? Test on heterogeneity?	Likelihood of publication bias assessed?	Conflict of interests stated?	Sum of items with ‘yes’
Homeopathy												
Perry 2010	No	Yes	Yes	No	No	Yes	Yes	Yes	Yes	No	No	6
Boehm 2014	No	Yes	Yes	No	No	Yes	Yes	No	Yes	No	No	5
Acupuncture												
Mayhew 2007	No	Cannot answer	No	No	No	No	Yes	Yes	Yes	No	No	3
Daya 2007	No	No	No	No	No	Yes	Yes	Yes	Yes	No	No	4
Langhorst 2010	No	Yes	Yes	Yes	No	Yes	Yes	Yes	Yes	Yes	No	8
Martin-Sanchez 2009	No	Cannot answer	No	No	No	Yes	No	No	Yes	No	No	3
Cao 2013	No	Yes	Yes	No	No	Yes	Yes	Yes	Yes	Yes	No	7
Deare 2013	Yes	Yes	Yes	Yes	Yes	Yes	Yes	Yes	Yes	Yes	No	10
Yang 2014	No	Yes	Yes	No	No	No	Yes	Yes	Yes	Yes	No	6
Chiropractic												
Ernst 2009	No	No	No	Yes	No	No	Yes	Yes	No	No	No	3
Herbal medicine												
de Souza Nascimento 2013	No	Yes	Yes	No	No	No	Yes	Yes	No	No	No	4
Multiple CAM												
Holdcroft 2003	No	No	Yes	No	No	No	Yes	Yes	Yes	No	No	4
Baronowsky 2009	No	No^a^	Yes	No	No	No	Yes	Yes	No	No	No	3
Terhorst 2011, 2012	No	Yes	Yes	Yes	No	No	Yes	Yes	Cannot answer	No	No	5
De Silva 2010	No	Yes	Yes	No	No	No	Yes	Yes	Cannot answer	No	No	4

^a^Not in study selection

**Table 3:**
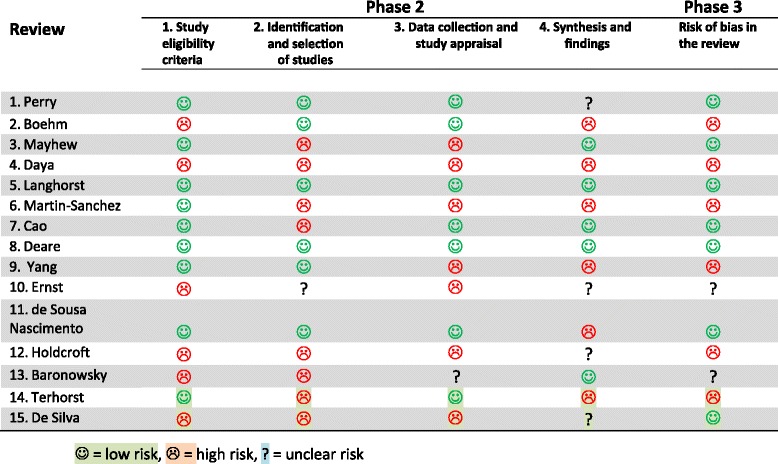
Tabular presentation for ROBIS results

The 15 included reviews were published between 2003 and 2014 and originated from seven countries. The included systematic reviews investigated the following therapies: homoeopathy (*n* = 2), acupuncture (*n* = 7), chiropractic (*n* = 1), herbal medicine (*n* = 1) and multiple CAMs (*n* = 4).

### Results of each CAM therapy

#### Homoeopathy

Two individual reviews of homoeopathy for FM were identified [28, 29]. Four multiple CAM reviews [30–34] also assessed homoeopathy. Perry et al. [28] included four RCTs [35–38] (three of which were placebo-controlled [35–37]). Their results suggested that homoeopathy was better than the control interventions in alleviating the symptoms of FM. However, none of the trials were without flaws. Using the Jadad scale [39] to assess the quality of the studies, two [35, 36] achieved a score of 3, one [37] achieved 4 and one [38] just 2 out of a possible 5. Blinding issues, small sample size, and lack of washout between crossover period were mentioned as some of the problems identified.

The review and meta-analysis by Boehm et al. [29] identified the same four RCTs and one controlled clinical trial (CCT) [40] (alongside ten case reports, three observational studies). A meta-analysis of three RCTs [36–38] (*n* = 139) revealed effects of homoeopathy on tender point count (SMD = −0.42; 95% CI −0.78 to −0.05, *P* = 0.03, *I* ^2^ = 0%), compared to placebo. Tender points are pain points or localised areas of tenderness around joints and are used to diagnose FM [41]. Also, a meta-analysis of two RCTs and one CCT [36, 38, 40] (*n* = 97) favoured homoeopathy in pain intensity using a 100-mm VAS (SMD = −0.54: 95% CI −0.97 to −0.10, *P* = 0.02; *I* ^2^ = 42%), compared to placebo. As this latter meta-analysis also included the results from the non-RCT, caution is needed in interpreting these results. Homoeopathy had no effect on the McGill Pain (MPQ) sensory scores (SMD = −0.08, 95% CI −0.51 to 0.34, *P* = 0.70, *I* ^2^ = 0%) when pooling two RCTs [37, 38]. Using the Cochrane Risk of Bias tool [42], two trials had a low risk of selection bias [37, 38], whilst the two randomised crossover trials [35, 36] did not report methods of randomization or allocation concealment. Only two trials reported adequate blinding of participants and personnel [36, 37], and all trials but one [40] reported adequate blinding of outcome assessment. Risk of attrition, reporting or other bias was low in most trials. Thus far, the effectiveness of homoeopathy as a symptomatic treatment for FM remains unproven.

### Acupuncture

We identified seven systematic reviews [43–49] that assessed acupuncture for FM alongside the four multiple CAM reviews [30–34]. One of the earlier reviews was conducted by Mayhew and Ernst [43] and included five RCTs [50–54] (*n* = 316) of various forms of acupuncture versus sham acupuncture (non-stimulation of acupuncture point or stimulation at traditional needle location). A meta-analysis was not performed, but the authors reported that three of the five included studies [51–53] found an effect of acupuncture. These effects were, however, mostly short-lived and, therefore, of debatable value [43]. Of the remaining two trials that did not favour acupuncture, one [54] was considered well designed and of good methodological quality using the Jadad scale [39].

Langhorst et al.’s [44] pooled analysis included seven studies [50–52, 54–57] (*n* = 242) and found strong evidence for the reduction of pain (SMD −0.25; 95% CI −0.49 to −0.02; *P* = 0.04, *I* ^2^ = 1%) at post-treatment compared to sham or simulated acupuncture. The methodological quality was assessed by the 11-item van Tulder score [58]). Sensitivity analyses demonstrated a significant effect on pain at post-treatment in studies with high risk of bias whereas the effect on pain at post-treatment in studies of low risk of bias did not demonstrate an effect.

Martin-Sanchez et al. [45] found, from a pooled analysis of four studies [51, 52, 54, 55] (*n* = 257), a SMD between acupuncture and sham groups from baseline of 0.02 (95% CI −0.24 to 0.28) with regard to pain intensity, but with wide confidence intervals which included the null value. Between-study homogeneity was in evidence (*P* = 0.41, *I* ^2^ = 0%) for this comparison. No assessment of quality or risk of bias was reported.

The meta-analyses conducted by Yang et al. (2014) [46] were inaccurate as they used the control group twice in the same analyses for two studies [53, 55]. Thus, we chose not to report the results from the meta-analyses here.

Cao et al. (2013) [47] found that acupuncture had no better effect than sham acupuncture with regard to pain relief in people with FM, as shown in pooled analysis of seven arms from five trials [52, 54, 55, 57, 59]. The change in VAS score was reported as SMD −0.09 (95% CI −0.32 to 0.14, *P* = 0.44 *I* ^2^ = 2%) and the VAS results at post-treatment were SMD −0.22, (95% CI −0.51 to 0.07, *P* = 0.13, *I* ^2^ = 26%). However, a pooled analysis of four trials [60–63] showed acupuncture was better than antidepressants in reducing VAS pain scores: SMD −0.60 (95% CI −0.93 to −0.27, *P* = 0.0004, *I* ^2^ = 22%). The small sample size, scarcity of studies for each comparison, and lack of an ideal sham weakens the level of evidence and its clinical implications. The only analyses we have reported here was that conducted on studies that compared acupuncture alone which did not incorporate mixed therapies in the meta-analyses. Two out of the 16 trials were evaluated as low risk of bias [55, 63], four [50, 54, 62, 63] were rated as having unclear risk of bias, whilst the other ten trials were evaluated as high risk of bias. Nine trials described randomization [51, 54, 55, 59–62, 64, 65], and six trials reported adequate allocation concealment [50, 51, 55, 59, 60, 64]. Three trials blinded both patients and outcome assessors [51, 55, 63]. Five trials reported the number of dropouts [51, 57, 63–65], and none of these trials used intention-to-treat analysis.

Deare et al.’s [48] Cochrane review identified eight RCTs and one quasi-RCT [66]. This is one of the most up-to-date systematic reviews on acupuncture. Pain severity (VAS 100 mm) showed a reduction in pain for those treated with real acupuncture compared with no acupuncture at the end of treatment (mean difference (MD) −22.40 points on a 100-point VAS scale; 95% CI −40.98 to −3.82, *P* = 0.02, favouring acupuncture). This was based on just one study [67]. Pain severity using pooled analysis of six studies of the VAS, numerical rating scale (NRS), the Westhaven Yale Multi-dimensional Pain Inventory (MPI) [68] and MPQ found no difference between groups in reducing pain ((*N* = 286) SMD −0.14; 95% CI −0.53 to 0.24, *P* = 0.48, *I* ^2^ = 54%). A short-term benefit of acupuncture over antidepressants was found in one study [66]; VAS = −17.3 on a 100-point scale; 95% CI −24.1 to −10.5. All studies except one were at low risk of selection bias; five were at risk of selective reporting bias (favouring either treatment group); two were subject to attrition bias (favouring acupuncture); three were subject to performance bias (favouring acupuncture) and one to detection bias (favouring acupuncture). Using the Grading of Recommendations Assessment, Development and Evaluation (GRADE) tool [42], they established there was low- to moderate-level evidence that compared with no treatment and standard therapy, acupuncture improved pain and stiffness in people with FM. There was moderate-level evidence that the effect of acupuncture does not differ from sham acupuncture in reducing pain. Electro-acupuncture was better than manual acupuncture for pain and stiffness reduction, although the effects were not maintained at 6 months follow-up.

#### Spinal manipulation (chiropractic/osteopathy)

There was just one review of chiropractic for FM consisting of three RCTs [69–71] and one quasi-RCT [72] conducted by Ernst in 2009 [73]. The reporting of the studies in this review was generally poor; just two reported statistical analysis of which neither found an effect of chiropractic treatment on pain. One quasi-experimental study [72] reported a 34% pain reduction versus 26% reduction in control group using a 100-mm VAS (but no further analysis was reported). However, both arms were in combination with exercise and drugs. All the trials were rated as low methodological quality according to the Jadad scale [39] (either scoring 1 or 2 out of 5). The current trial evidence is insufficient to conclude that chiropractic treatment is an effective treatment for FM.

Baronowsky et al.’s (2009) [31] review of multiple CAM therapies included one study of osteopathy [74] which reported that the osteopathy group did better than the control group in pain threshold in three tender points; again, analysis was not reported.

#### Herbal medicine

There was just one review on herbal medicine for FM conducted in 2013 by de Souza Nascimento [75]. This review reported on eight studies of different types of herbal medicine. One multiple CAM review also assessed herbal medicine [32]. The results from both these reviews vary depending on which herbal extract is used. No meta-analysis was conducted due to heterogeneity of the interventions.

One study [76] using topical *Capsicum* (chilli pepper) found an improvement in myalgia score, pressure pain threshold, and Fibromyalgia Impact Questionnaire (FIQ). Another study [77] found an improvement in sensitivity and pain. Size of effects/*P* values were not reported in either study. One RCT [78] found that *nabilone* was similar to *amitriptyline* on pain scores and one [79] found a decrease in pain in nabilone group. Again, actual results were not reported.

024-oil pain neutralizer, which contains *camphor*, *eucalyptus oil*, *aloe vera oil*, *peppermint oil*, *lemon* and *orange oil*, was investigated in two studies [80, 81]. Only one [80] reported on pain and found an improvement in night pain rating and tender point count. *Meta-050* (a combination of reduced iso-alpha-acids from hops, rosemary, and oleanolic acid) was also only assessed in one open study [82] and found after 8 weeks, both pain and stiffness were moderately improved. The methodological quality of all included trials was evaluated by using Jadad scale [39] and two studies were rated as good quality [76, 79], four studies moderate [77, 78, 80, 81] and two studies low [82, 83]. In addition, risk of bias was assessed by the Cochrane Risk of Bias tool. Most studies were low for section bias. Five of eight studies were double-blind [77–81]; thus, these studies had a low risk of performance bias and low detection bias. No detailed evidence of selective reporting was found in any of the eight studies.

#### Multiple CAM Reviews

Four systematic reviews [30–34] assessed several CAM therapies within the same paper. We were only interested in some of these therapies, so we have selected the CAMs that were relevant to our review objectives and reported them in the relevant sections above.

#### Adverse events

Poor reporting of adverse events (AEs) is a frequent criticism of CAM research [84]. However, nine [30, 32, 43, 44, 46–49, 75] of the 15 reviews report on adverse events. A range of adverse events were reported, depending on which CAM was utilised. With regard to acupuncture, AEs were often either exacerbations of existing symptoms or unpleasantness of the intervention itself. Mild bruising, soreness, typically discomfort at site of needle and nausea were reported. In contrast, palpitations, fainting, dry mouth, fatigue and constipation were AEs associated with anti-depressant medication that was used as treatment as usual in some groups. De Silva et al. [32] found that in one homoeopathic study, allergic reactions were reported. AEs were well reported in de Souza Nascimento et al’s. [75] review of herbal remedies. Transient, burning and pricking, skin irritation, dizziness, nausea, dry mouth, drowsiness, constipation and insomnia were some of the side effects associated with herbal medicines.

#### Quality of included reviews

##### Results of AMSTAR

A summary of the AMSTAR results can be found in Table 2. Nine reviews reported using two data extractors and achieving study consensus. Just one review did not report conducting a risk of bias assessment [45], and two [29, 45] did not apply the quality assessment appropriately in light of the findings. Only one included an ‘excluded studies’ table [48]. Seven reviews [28, 29, 44, 45, 47–49] included detailed characteristics of the included studies; the majority had some form of table, but not every review reported on participant details. Details on the intervention and outcomes were generally better reported in most reviews. The methods used to combine the studies were reported and appropriate in 11 reviews. Four assessed likelihood of publication bias (through funnel plots) [44, 46, 48, 53]. None of the reviews stated conflict of interest of the individual studies. Overall, five reviews scored 6 or above on the AMSTAR scale [28, 44, 46–48]. The inter-rater agreement was good (*Ƙ* = 0.70), with 83.6% agreement between the two raters (RP, VL).

##### Results of ROBIS

The ROBIS tool is divided into four domains (see Table 3 for summary of results and Appendix 1 for full results). With regard to domain 1, which assessed any concerns regarding specification of study eligibility criteria, nine reviews [28, 33, 34, 43–48, 75] achieved a low risk of bias rating overall. Domain 3 assessed concerns regarding methods used to collect data and appraise studies, and seven studies achieved a low risk of bias rating [28, 29, 33, 34, 44, 47, 48, 75]. With regard to domain 4, which assessed concerns regarding the synthesis and findings, there was more variation in the scores; six were assessed as high [29, 33, 34, 45, 46, 49, 75], four unclear [28, 30, 32, 73] and five scored low [31, 43, 44, 47, 48]. The reviews that did not conduct a meta-analysis were hard to assess using ROBIS. The final section provides a rating for the overall risk of bias of the reviews; seven achieved a low rating [28, 32, 43, 44, 47, 48, 75], six a high rating [29, 30, 33, 34, 45, 46, 49] and two were rated as unclear [31, 73]. The inter-rater agreement was fair (*Ƙ* = 0.32), with 60.0% agreement between the two raters (RP, VL).

## Discussion

### Summary of the main results

#### Homoeopathy

Two individual reviews and four multiple CAM reviews assessed homoeopathy for FM. The most recent review [29] included the same RCTs as Perry et al. [28] but also included 13 observational studies. This achieved 5/11 on Amstar and was considered high risk of bias by ROBIS. Perry et al. [28] was a more robust review with a low risk of bias rating by ROBIS and scoring 6 (high quality) on AMSTAR. Although there was some positive effects demonstrated, more research is needed before homoeopathy can be considered a viable alternative treatment for FM.

#### Acupuncture

From the seven acupuncture reviews and four multiple CAM reviews, the most robust evidence regarding acupuncture comes from Deare et al.’s [48] Cochrane review. This achieved a positive response on 10/11 on the AMSTAR components and was judged to be of low risk of bias on each of the five ROBIS domains. They concluded that there was low-quality evidence that acupuncture improves pain compared to no treatment or standard treatment, but good quality evidence that it is no better than sham acupuncture. This is an interesting and unexpected result as it implies that acupuncture is equivalent to placebo but more effective than standard care (antidepressants). However, the sham conditions varied from sham needling to acupuncture in a non-acupuncture place, which might indicate there were blinding issues in some of these sham groups. Alternatively, it could indicate there is a genuine placebo response to sham acupuncture. As this is one of the most recent and robust reviews, its conclusions carry more weight than the other reviews on acupuncture.

#### Spinal manipulation

One review of chiropractic [73] was identified and scored 3/11 on AMSTAR and assessed as high risk of bias on ROBIS. There were several problems with the individual RCTs; thus, the results were inconclusive. One multiple CAM review [31] assessed osteopathy and indicated the results favour osteopathy over standard care alone. However, this review was rated as unclear on ROBIS and scored 3 on AMSTAR.

#### Herbal medicine

The one herbal medicine review [75] and one multiple CAM review [32] both indicated some evidence for topical *Capsicum.* 024-oil and nabilone also reported positive results for pain. However, as nabilone is made up of cannabinoid extract, it may not be considered a preferred treatment option for some people with FM. de Souza Nascimento et al. [75] only scored 4/11 on AMSTAR but achieved a low risk of bias score when using ROBIS which indicates different interpretations/assessments of quality when using the two tools.

### Overall completeness and applicability of evidence

With regard to the eight CAMs we were interested in, our overview is in agreement with Lauche et al.’s [24] work which suggested that acupuncture had the best evidence of effectiveness for FM, conflicting results for chiropractic and inconclusive results for homoeopathy and phytotherapy (herbal medicine). In addition, some reviews that we identified were missing from Lauche’s overview [29, 46, 47, 49]. It is unclear from their inclusion/exclusion criteria why these four reviews would have been excluded. Thus, our overview provides a more up-to-date overview of the selected CAMs.

Our overview also drew similar conclusions to Terry et al.’s [1]. They also found some evidence of beneficial effects arising from both acupuncture and homoeopathy for the treatment of FM symptoms, whilst no evidence for therapeutic effects from chiropractic interventions was found.

### Quality of the evidence

To date, AMSTAR is one of the main scales for assessing quality of a systematic review. It is quick and easy to complete, and there was good inter-rater reliability (kappa = 0.70, agreement 83.6%). In general, there was consistency between ROBIS and AMSTAR. Five reviews [28, 44, 46–48] achieved a high overall rating (scores >6) with the AMSTAR scale (although AMSTAR is not designed to have a final score). These five reviews also all scored low risk of bias on ROBIS. There were discrepancies on rating for three reviews; Mayhew and Ernst [43] achieved a low risk of bias but scored just 3 on AMSTAR, Yang et al. [46] achieved a high risk of bias but scored 6 on AMSTAR and de Souza Nascimento et al. [75] achieved a low risk of bias score on ROBIS but scored 4 on AMSTAR.

If a meta-analysis was included, this made rating domain 4 of the ROBIS tool easier to complete. Narrative syntheses were much harder to rate on this particular domain. There is little information in the ROBIS guidance document on how to score the signalling questions where no quantitative synthesis has been done or where the small number of studies included in the quantitative synthesis does not permit exploration of the data with regard to heterogeneity, robustness of the finding and quality. De Silva et al. [32] was an interesting review. Despite scoring high or unclear for domains 1–4 they still achieved a low score overall; this was because they did not overemphasise their findings and were able to critique their shortcomings of the review process. This highlights one of the strengths of ROBIS.

### Potential bias in the overview process

One author evaluated their own work [RP: 28] and one of the developers of ROBIS (PD) was involved in the applying ROBIS to assess the included reviews. Another of the developers of ROBIS (RC) was involved in the write up of the report. Although the search strategy was comprehensive, it is possible that some relevant reviews may not have been identified. In addition, a limitation of the overview is that several of the included reviews would be considered out of date (more than 5.5 years) [85]. Some reviews were excluded, due to language restrictions we imposed. This was due to requiring a trained person in the ROBIS tool to complete the assessment. This meant two potential reviews were excluded due to language [86, 87] (see Table 4 in Appendix 2). Despite these issues we believe the systematic approach to this overview minimises bias. Difficulties in using ROBIS may have led to errors in interpretation; lower inter-rater reliability was achieved than when using AMSTAR. In addition, CAM papers tend to be published in lower impact journals and often restricted by word count. Earlier reviews did not tend to score so highly on either tool probably because reporting criteria have changed over time.

## Conclusions

### Authors’ conclusions

#### Implications for CAM practice

Of all the CAM interventions included, acupuncture received the most positive assessment in terms of effectiveness. This was the conclusion from the most recent Cochrane review [48]. This review was rated as good quality using AMSTAR and low risk of bias using ROBIS. Further well-conducted trials on herbal extracts such as *Capsicum*, nabilone and 0il-24 would also be beneficial.

#### Implications for future research

There is clearly a need for larger and more methodologically sound RCTs to be conducted on the effectiveness of some CAM therapies for FM. Acupuncture, in particular, had several trials investigating its efficacy for FM. Future trials could adopt the following RCT design: to compare drug plus sham acupuncture versus placebo drug plus CAM intervention. This would enable the sham condition to be tested properly.

Both reviews assessing herbal medicine [32, 75] indicated some evidence for topical *Capsicum* but more research is needed. More research is also needed before homoeopathy can be considered a viable alternative treatment for FM.

### Overall conclusions

Overall, no firm conclusions were drawn for either spinal manipulation or homoeopathy for FM. There is limited evidence for topical *Capsicum* to alleviate symptoms of FM, but more research is needed. There is some evidence to support the effectiveness of acupuncture for FM, and further high-quality trials are needed to investigate its benefits, harms and mechanisms of action, compared with no or standard treatment before this can be considered a viable alternative treatment for FM.

## Appendix 1

### Description of CAM therapies

Acupuncture is the insertion of the tips of needles into the skin at specific points for the purpose of treating various disorders by stimulating nerve impulses. Originally Chinese, this method of treatment is practised in many parts of the world [88]. It aims to restore balance to enable the chi to free flow around the meridians. Each meridian is associated with a particular organ [89]. Western medical acupuncture has evolved from these ideas and is more about stimulating the nervous system (http://www.nhs.uk/Conditions/hypnotherapy/Pages/Introduction.aspx). Acupuncture is one of the more established CAM therapies within primary care, and it is an important CAM to review [21].

Hypnotherapy is a form of induced sleep which was originally used to diminish pain during surgery but soon became redundant with the advent of anaesthesia [90]. It is used to create subconscious change in a patient in the form of new responses, thoughts, attitudes, behaviours or feelings. It is often used in treating anxiety states, stopping addictions and reducing pain [91].

Homoeopathy is based on the principle of like cures like [92]. The remedies are prepared by dilution and energised through succession. Several aspects of the treatment (e.g. long, empathetic consultation and a high degree of individualising the remedies) might make it particularly attractive to patients with FM [93].

Osteopathy is a way of detecting, treating and preventing health problems by moving, stretching and massaging a person’s muscles and joints (http://www.nhs.uk/conditions/Osteopathy/Pages/Introduction.aspx).

Chiropractors uses less leverage and quicker manipulations than osteopathy, also uses soft tissue massage, exercise, corsets, splints and supports (http://www.nhs.uk/conditions/chiropractic/pages/introduction.aspx). The mechanical technique of either form of spinal manipulation might make it less attractive to FM sufferers as the pain tends to be throughout the body and manipulation may worsen this pain.

Herbal medicine is the use of plant extracts/materials for food medicine and health promotion. Medicinal plants have multiple actions; some of which are toxic. As humans, we are raised in a diet of herbs and plants so the suggestion is that we are better adapted to them than synthetic drugs. The plants are used in a variety of ways: dried fresh, infusion or decoctions [88].

Reflexology is a specialist foot massage which concentrates on specific zones on your foot relating them to major organ systems within the body. Blocks or disturbances within the connecting energy system allow for disease to build up, and these channels need unblocking [94].

Aromatherapy is the use of essential oil or aromatic essences massage into the skin, inhaled or occasionally ingested. The oils are extracted from the petals, leaves, stem or bark of the plant [95].

## Appendix 2

**Table 4:** Excluded reviews

Author (date)	Reason for exclusion
Berman BM [105]	Not a systematic review
Schneider M [106]	Consensus report
Sim J [107]	Multiple CAM review with just one relevant study included
Langhorst J [86]	German language (needed translation)
Lauche [87]	German language (needed translation)
Hardy-Pickering [108]	Overview of systematic reviews (conducted in 2007—so considered out of date)

**Table 5:** Table of reviews in progress

Boyd A [109]	Herbal medicinal products or preparations for neuropathic pain and fibromyalgia PROTOCOL (Cochrane review) At August 2016, this protocol was withdrawn due to the full review not meeting the quality standards and expectations of Cochrane and the PaPaS review group.
Jones GT [110]	Published as part of a report: Arthritis Research UK—A report on Complementary and alternative therapies. ‘Practitioner-based CAM for the treatment of rheumatoid arthritis, osteoarthritis, FM and low back pain.’

## Appendix 3

### MEDLINE search terms

1. systematic review.ti,ab.

2. meta-analysis.pt.

3. meta-analysis.ti,ab.

4. systematic literature review.ti,ab.

5. review.pt.

6. evidence synthesis.ti,ab.

7. 1 or 2 or 3 or 4 or 5 or 6

8. exp Fibromyalgia/

9. (chronic adj widespread adj pain).ti,ab.

10. fibrositis.ti,ab.

11. fibromyal*.ti,ab.

12. fibromylagia.ti,ab.

13. 8 or 9 or 10 or 11 or 12

14. homeopathy.ti,ab.

15. homeopathic.ti,ab.

16. homeop*.ti,ab.

17. homoeopathy.ti,ab.

18. homoeopath*.ti,ab.

19. homoop*.ti,ab.

20. exp Homeopathy/

21. 14 or 15 or 16 or 17 or 18 or 19 or 20

22. acupuncture therapy.ti,ab.

23. electroacupuncture.ti,ab.

24. acupuncture*.ti,ab.

25. acupoint.ti,ab.

26. meridian.ti,ab.

27. moxibustion.ti,ab.

28. exp acupuncture/

29. 22 or 23 or 24 or 25 or 26 or 27 or 28

30. (spin* adj3 manipulation*).ti,ab.

31. (osteopath* adj manipul*).ti,ab.

32. (high adj3 velocit* thrust).ti,ab.

33. (spin* adj3 adjust*).ti,ab.

34. (sham adj3 manipulation*).ti,ab.

35. exp Manipulation, Chiropractic/

36. exp Manipulation, Spinal/

37. exp Manipulation, Osteopathic/

38. chiropract*.ti,ab.

39. osteopath*.ti,ab.

40. 30 or 31 or 32 or 33 or 34 or 35 or 36 or 37 or 38 or 39

41. exp Hypnosis/

42. (hypno* or autogenic* or mesmer* or guided ima*).ti,ab.

43. 41 or 42

44. reflexolog*.ti,ab.

45. reflexolog* treatment*.ti,ab.

46. foot massage*.ti,ab.

47. zone therap*.ti,ab.

48. 44 or 45 or 46 or 47

49. (herbal* or medical herbal* or TCM).ti,ab.

50. exp Drugs, Chinese Herbal/

51. exp Phytotherapy/

52. 49 or 50 or 51

57. 13 or 21 or 29 or 40 or 43 or 48 or 52

58. 7 and 13 and 57

## Appendix 4

**Table 6:** Summary of the ROBIS domains

Review	1. Study eligibility criteria	2. Identification and selection of studies	3. Data collection and study appraisal	4. Synthesis and findings	5. Risk of bias in the review
Homeopathy					
Perry (2010)	Low: There was no mention of a review protocol but did mention that the inclusion/exclusion criteria were pre-defined. Some additional searching took place; reference lists and other reviews were hand-searched.	Low**:** Although the search included appropriate databases to identify published studies, searches did not included trial registries or conference reports. The review was restricted to published studies. Two reviewers looked at full texts, but this was not specifically stated for abstract screening.	Low**:** Two reviewers independently performed data extraction and risk of bias assessment. Risk of bias was assessed using appropriate criteria (Jadad score [39]) with allocation concealment being assessed in addition. Some study characteristics were extracted (main table), but information was missing on participants. Appropriate results appear to have been collected although this is not completely clear.	Unclear: There was heterogeneity; thus, no meta-analysis was performed. Each study was discussed and evaluated in detail, and a sufficient synthesis occurred. The results of the risk of bias assessment were reported in full. This narrative review assesses the results appropriately and the conclusion reflects this.	Low: The main concerns arising from this were the potential for missed studies through not includ unpublished papers. The conclusions seem fair in relation to these considerations.
Boehm (2014)	High: There was no mention of a review protocol or pre-specification of review objective. There were some concerns regarding the specification of the eligibility criteria with regard to diagnosis of fibromyalgia. No specific list of outcomes stated.	Low: Although the search included appropriate databases to identify published studies, searches did not included trial registries or conference reports. Limited details on the search strategy. The term ‘homeopathy’ was used which would not pick up ‘homeopathic’.	Low**:** Two reviewers independently performed data extraction and risk of bias assessment. Risk of bias was assessed using appropriate criteria (Cochrane risk of bias [42]). Appropriate study characteristics were extracted, and appropriate results appear to have been collected.	High**:** One Fisher study (1986) was not included in the synthesis, but it unclear why it was excluded. Combining RCTs with non-RCTs will introduce bias.	High**:** The discussion is mostly cautious although the final sentence is a bit over-confident. Some attention given to inclusion of different study designs and the ambiguous definition of homeopathic remedy.
Acupuncture					
Mayhew (2007)	Low: There was no mention of a review protocol or pre-specification of review objective. There was some concern regarding the specification of the eligibility criteria with regard to outcomes as no outcomes were mentioned.	High: Although the search included appropriate databases to identify published studies, searches did not included trial registries or conference reports. Limited details were available for the search strategy; the full search was not reported. Methods used to screen references and select studies for inclusion were not reported.	High: Two reviewers independently performed data extraction. It was unclear if the two assessed risk of bias. Risk of bias was assessed using appropriate criteria (Jadad score [39]) although allocation concealment is not assessed. There was some reporting of means and percentage differences between groups but not for every study. They also failed to define outcome at the start.	Low: There was limited result information given and as there was no protocol; we cannot check outcomes that were intended to be assessed. This is not really a synthesis, more like a list of finings.	Low: Although some of the domains had issues, the conclusion does take into account some of the weaknesses of the studies and does not overemphasise any positive findings.
Daya (2007)	High: There was no mention of a review protocol or pre-specification of review objective. Lack of detail on eligibility criteria and limited to English language.	High: Although the search included appropriate databases to identify published studies, searches did not include trial registries or conference reports. Limited details were available for the search strategy (no mention of MeSH headings). It appears that the review was restricted to published studies. Methods used to screen references and select studies for inclusion were not clearly reported and appeared to be done by just the author, so no cross-checking.	High: One reviewer performed data extraction and risk of bias assessment. Risk of bias was assessed using appropriate criteria (Stricta [104]). Appropriate study characteristics were extracted (main table) but only *P* values appear to have been extracted.	High**:** The results of the individual studies are reported without any real attempt at a synthesis. The quality scale also includes other items which is likely to affect the overall score. Conflicting results between the highest quality studies suggests the findings were not robust.	High**:** The conclusions seem appropriate for the limitations of the evidence. Main concerns are the potential for missing studies from the limited search and mainly due to a single person conducting the review with no cross-checking.
Langhorst (2010)	Low: There was no mention of a review protocol or pre-specification of review objective. However, there were very detailed eligibility criteria. The search was restricted to fully published studies. The type of acupuncture was restricted to ‘verum’ acupuncture (inserting needles). Acupressure, TENS, and infrared light were excluded, which are appropriate exclusions.	Low**:** Although the search included appropriate databases to identify published studies, searches did not included trial registries or conference reports. Reference lists, other systematic reviews, and evidence-based guidelines were also searched. The search looks reasonable and is transparent. Methods used to screen references and select studies for inclusion were clearly reported.	Low: Two reviewers extracted data, but it does not state directly in the text if two reviewers independently performed risk of bias assessment (van Tulder score [58]). Risk of bias was assessed using appropriate criteria. Appropriate study characteristics were extracted (main table).	Low**:** There is a slight error in reporting of results in text and in forest plots. Publication bias could not be assessed due to low number of studies. Sensitivity analysis looked at those with low risk of bias did not show an effect in the meta-analysis.	Low**:** Main concerns arising from this review were the potential for publication bias though only including published studies. It did not clearly state whether two people assessed risk of bias. However, the analysis and sensitivity analysis were appropriate and thorough and helped the authors draw more conservative and appropriate conclusion.
Martin-Sanchez (2009)	Low**:** There was no mention of a review protocol or pre-specification of review objective. Inclusion criteria were brief but there did not appear to have any restrictions.	High**:** Although the search included appropriate databases to identify published studies, searches did not include trial registries or conference reports. Limited details were available for the search strategy. No MeSH terms were mentioned, and full search was not reported. They did not search any CAM databases. Limited number of references identified. There was no information on restrictions e.g. date, publication format, language. Methods used to screen references and select studies for inclusion were not clearly reported.	High**:** There was insufficient reporting on all aspects of data collection, risk of bias assessment and results*.*	High: It was unclear why studies were not included in the meta-analysis. The first meta-analysis consisted of 4 of 6 studies. Heterogeneity was discussed briefly. There was no quality assessment, so no insight into methodological quality or risk of bias. No sensitivity analysis.	High: None of the limitations identified were considered in the discussion. Think it is highly likely that reviewers have missed studies. No consideration of study quality, which is a key component of systematic reviews.
Deare (2013)	Low: Cochrane reviews are required to have a protocol which is peer assessed before the review can commence. No restrictions on language and publication type. There were restrictions in studies that did not provide adequate details of control group. Conference abstracts appear to be excluded (see flow diagram).	Low: There were no major concerns with this section. It appears just one reviewer did the screening of titles and abstracts though.	Low: No concerns with this section. Two reviewers independently performed data extraction and risk of bias assessment. Risk of bias was assessed using appropriate criteria (Cochrane [42]). Appropriate study characteristics were extracted (main table), and appropriate results appear to have been collected.	Low**:** No major concerns; however, one thing to highlight is concerning robustness of the findings. This judgment depends on the comparison: Acupuncture V no acupuncture (just 1 study) Acupuncture V placebo/sham (robust findings)	Low**:** The conclusion was appropriate and addressed the concerns raised.
Cao (2013)	Low: There was no mention of a review protocol or pre-specification of review objective. However, outcomes were not clearly specified and did not appear to constitute an objective pre-specified list.	High: Although the search included appropriate databases to identify published studies, searches did not included trial registries or conference reports. The search strategy appeared comprehensive, but it was unclear if both MeSH and text word used. It appears that the review was restricted to published studies although this was not completely clear. Methods used to screen references and select studies for inclusion were clearly reported.	Low**:** Two reviewers independently performed data extraction and risk of bias assessment. Risk of bias was assessed using appropriate criteria (Cochrane ROB [42]). Appropriate study characteristics were extracted (main table), and appropriate results appear to have been collected although this is not completely clear.	Low**:** Unclear if MA included all suitable papers. Lack of guidance on ROBIS tool about how to appropriately consider robustness of quality on results when there is insufficient numbers of studies.	Low: The conclusion seemed to address all the concerns raised in the other domains.
Yang (2014)	Low**:** There was no mention of a protocol, but there was detailed pre-specification of review objectives. Over all, there were limited concerns with this domain but the texts were restricted to Chinese and English which should be ok for an acupuncture review.	Low: Although the search included appropriate databases to identify published studies, searches did not include trial registries or conference abstracts. Methods used to screen and select studies for inclusion were clearly reported. The language restriction has been dealt with in domain 1.	High**:** Risk of bias was assessed using Cochrane criteria; however, both Harris (2005) and Guo (2005) have been assessed twice and have very different risk of bias scores despite being the same study. This is confusing and questions whether errors have been made in the assessment.	High**:** In the meta-analysis, the reviewers could have synthesised the VAS and NRS in the same forest plot. Again, there is an issue of Harris and Guo appearing twice in the plots even though they are the same study (with the same control arm). Results are not robust as there are insufficient studies to assess robustness.	High**:** ‘Despite the methodological limitations the superiority of acupuncture in the treatment of FMD cannot be denied’ is an overstatement. The flaws in the assessment of risk of bias and the untrustworthy results from the meta-analysis make this review of high risk of bias.
Chiropractic					
Ernst (2009)	High**:** There was no mention of a review protocol but did mention that the inclusion/exclusion criteria were pre-defined. No mention of patients with a formal diagnosis of fibromyalgia.	Unclear**:** Although the search included appropriate databases to identify published studies, searches did not included trial registries or conference reports. Departmental files were searched (which could be a biased selection) and hand-searching took place. The full search strategy was not provided. Methods used to screen references and select studies for inclusion were not clearly reported. It was not reported how many reviewers screened titles and abstracts.	High: Two reviewers independently performed data extraction and risk of bias assessment. Risk of bias was assessed using appropriate criteria (Jadad score [39]) although allocation concealment was not assessed. Some study characteristics were extracted (main table), but information was missing on participants. Appropriate results (when available) appear to have been collected although this is not completely clear.	Unclear: The results of the risk of bias assessment were reported in full; however, allocation concealment was not assessed. This narrative review assesses the results available; however, no numerical results given. Heterogeneity was not formally assessed. The results from Wise and Walsh were not reported in the primary study; thus, a possible source of bias as their results could affect the overall conclusions.	Unclear**:** The conclusions are inconclusive which is reasonable based on the evidence available. The possibility of missing studies is discussed. The studies are rated low quality so item on allocation concealment is unlikely to have changed this (Jadad scale).
Herbal medicine					
de Souza Nascimento (2013)	Low: The review did not refer to a protocol; however, the inclusion/exclusion criteria were pre-defined. The review was restricted to English language papers only. Not much grey literature searching took place.	Low**:** The search included appropriate databases to identify published studies. Reference lists were hand-searched. The search looks reasonable and is transparent, although CAM-specific databases were not searched. It is unclear whether unpublished papers would be identified. It appears that the review was restricted to published studies. Trial registries were not searched. Methods used to screen references and select studies for inclusion were not clearly reported.	Low**:** It states in the text that two reviewers independently performed risk of bias assessment. Risk of bias was assessed using appropriate criteria (both Jadad and Cochane). Insufficient study characteristics were extracted, and there was not enough information about the actual results obtained—just ‘a significant difference was found.’ No actual data provided, just a summary of the result. Unclear which results were used to come to these conclusions. A pilot study was mentioned (Triaste) but no further information as to why this was excluded.	High: No protocol provided Heterogeneity not discussed. It was unclear why certain studies could not be combined. Narrative synthesis of results mentioned the direction of effect but no information about the size of the effect.	Low*:* Main concerns arising from this were the potential for publication bias through only including published studies and restricting to English language only. There was a tendency to emphasise the positive findings. They made no mention of the small number of studies or the risk of bias in those studies when interpreting the results.
Multiple CAM					
Holdcroft (2003)	High**:** There was no mention of a review protocol and ambiguous eligibility criteria. There were no fibromyalgia criteria or any outcomes listed. One restriction was to only include those studies judged as good quality of reporting.	High**:** Although the search included appropriate databases to identify published studies; searches did not included trial registries or conference reports. Search strategy was not available and they have not put the term for homeopathy in (although they do retrieve one study on homeopathy). There are odd search dates for Embase and CINAHL which restrict the search. There is no information about study selection.	High: There was no information about participants or how outcomes were measured. Little information about dose and nothing on study design. One reviewer performed data extraction and quality assessment. The CONSORT checklist was used as a quality assessment tool which is inappropriate. No results are displayed in the table or results section just a statement ‘differed significantly’.	Unclear**:** No numerical synthesis due to heterogeneity; there was no flow diagram and no list of included outcomes, so it is unclear whether results of studies were not included that should have been (possibility of ‘cherry-picking’ the results). Study design and quality is considered in the narrative; however, the CONSORT checklist is an inappropriate scale to assess quality.	High**:** The conclusion was suitably cautious but no mention of limitations identified in domains 1-3.
Baronowsky (2009)	High**:** There was no mention of a review protocol or pre-specification of review objectives. No mention of comparators or outcome of interest. Articles were restricted to English and German languages only which may have missed some papers (particularly Chinese). Nutritional, Herbal medicine and hormonal supplements were excluded from the review.	High: Although the search included appropriate databases to identify published papers, and the terms appears to cover all the CAM therapies that were needed (although no MeSH terms listed), it appears this restricted to published papers. Studies are likely to have been missed due to not searching beyond electronic databases. Details of the screening process were not clearly reported.	Unclear: Quite a few items on the quality assessment checklist are not about quality so this will affect the score. Also, it is not clear how many people assessed quality. Limited information reported on participants. Insufficient results are presented, and actual results (means, SDs) were not reported. *P* values were reported occasionally within a statement mentioning significance.	Low: There was insufficient reporting of outcomes evaluated and the numerical results. This is particularly an issue when there is no meta-analysis available.	Unclear**:** Overall, the results show a positive trend in favour of acupuncture. Which might be overstating the findings a bit. The possibility of missing studies is discussed however.
De Silva (2010)	High: There was no mention of a review protocol and a limited pre-specification of review objective. Inclusion was restricted to studies a complementary medicine substance in the UK which restricts this review. It was also restricted to English language.	High**:** Although the search included appropriate databases to identify published studies, limited details were available for the search strategy. The RCT filter was very basic and likely to miss some trials. It appears that the review was restricted to published studies although this was not completely clear. The search was restricted to electronic databases. Only 60 references were identified which seems quite limited. Methods used to screen references and select studies for inclusion were clearly reported.	High: Not all data was provided, e.g. results of some studies were not reported. Some *P* values reported in text. No information in the methods section about results data to be collected. One reviewer performed data extraction and risk of bias assessment, and this was checked. Risk of bias was assessed using Jadad only which is a limitation (no allocation concealment).	Unclear**:** They seemed to have reported the same number of results as number of studies although not all *P* values given. No pre-defined analysis. No description of outcomes of interest given in the paper so impossible to judge whether the paper should be included or not. No mention of heterogeneity but no meta-analysis completed so assumed this was an issue. Only a single trial for some CAMs, so no synthesis of any type was possible. Restricting to CAM administered just in the UK restricts the generalizability of the results.	Low: Rationale for risk: the small number of studies, methodological limitations and limiting the search to English language only. Use of the Jadad scale was another issue. However, the conclusion does say there is insufficient evidence available.
Terhorst (2011, 2012)	Low: There was no mention of a review protocol and a limited pre-specification of review objectives. In CAM research, there are often lots of Chinese papers, so excluding non-English papers is risky.	High: Although the search included appropriate databases to identify published studies, there were limited details available on the search strategy. There was a broad range of sources of references searched; searches also included dissertations. Methods used to screen references and select studies for inclusion were reported.	Low**:** Unclear if two reviewers extracted data although it states that two assessed risk of bias. Risk of bias was assessed using appropriate criteria (Cochrane GRADE [42]) although adaptions were made. For a review of this size, appropriate study characteristics were extracted (main table) and appropriate results appear to have been collected.	High: Meta-analysis did not include a sensitivity analysis based on quality. Heterogeneity was not assessed. Studies excluded from the analysis were explained. They pooled the data but did not report on how they combined the effect sizes. There was no synthesis in categories where there were less than 5 studies.	High: There was a tendency to be over-positive about the results in general considering the limitations of the search and restricting to English language only limits this review.

## Abbreviations

ACR: American College of Rheumatology
AMSTAR: Assessing the methodological quality of systematic reviews
CAM: Complementary and alternative medicine
CCT: Controlled clinical trial
CI: Confidence interval
FM: Fibromyalgia
GRADE: Grading of Recommendations Assessment, Development and Evaluation
MD: Mean difference
MPQ: McGill Pain Questionnaire
RCT: Randomised controlled trial
SD: Standard deviation
SIGN: Scottish Intercollegiate Guidelines Network
SMD: Standard mean difference
SS: Symptom severity scale
TCM: Traditional Chinese medicine
VAS: Visual analogue scale
WPI: Widespread Pain Index
